# Inter-Examiner Agreement of the National Upper Cervical Chiropractic Association Analysis of the Atlas Subluxation Complex in a 3-View Upper Cervical Radiographic Series

**DOI:** 10.1016/j.jcm.2023.04.001

**Published:** 2023-07-14

**Authors:** Jordan Landholm-Duvall, D. Gordon Hasick, Harrison Ndetan, John F. Hart, Marshall Dickholtz, Craig P. Lapenski

**Affiliations:** a Upper Cervical Research Foundation, Minneapolis, Minnesota; b Department of Epidemiology and Biostatistics, University of Texas Health Science Center, Tyler, Texas; c Hart Research Consulting, Greenville, South Carolina

**Keywords:** Reproducibility of Results, Diagnostic Imaging, Chiropractic, Radiography, Cervical Vertebrae

## Abstract

**Objective:**

The purpose of this study was to measure the inter-examiner agreement between radiograph markings of 2 National Upper Cervical Chiropractic Association board-certified chiropractors.

**Methods:**

Two chiropractic examiners who had standardized training marked and analyzed 254 conventional orthogonal radiographic film sets. The level of agreement and potential biases in their measurements were assessed using intraclass correlation coefficients for absolute agreement and Bland-Altman plot analyses.

**Results:**

There was 96.1% agreement between the examiners in the measurements of the side of atlas laterality and 94.5% for atlas rotation. The intraclass correlation coefficient was 0.95 (95% CI, 0.93-0.96) for atlas laterality and 0.92 (95% CI, 0.89-0.94) for atlas rotation. The mean difference in the measurement between the 2 examiners was −0.11, *P* = .12 for atlas laterality and 0.05, *P* = .55 for atlas rotation. Neither atlas laterality nor atlas rotation measurements were significantly different from zero. Bland-Altman plots were not suggestive of any proportional biases in the 2 measurements.

**Conclusion:**

Results of this study show almost perfect agreement between 2 trained chiropractic examiners, with no apparent proportional bias in the analysis of conventional orthogonal radiographic film sets.

## Introduction

There are several studies that have looked at chiropractic doctors’ ability to reach inter-examiner agreement when analyzing cervical spine radiographs.^1^, ^2^, ^3^, ^4^, ^5^, ^6^ A 1985 study observed large ranges of error in analyses, concluding, “any measured differences produced using this system will be just as likely to be from marking error as from actual atlas position change.”^1^ Another study concluded, “the side of atlas laterality (right or left) is in 100% agreement in this study.”^2^ Other similar studies concluded adequate reliability in their analysis systems.^3^, ^4^, ^5^, ^6^ While these studies contributed to the body of literature, none had a large enough sample size or specifically addressed the reliability between doctors utilizing the National Upper Cervical Chiropractic Association (NUCCA) radiographic analysis protocol.

Radiographic analysis is a required skill set for upper cervical chiropractic doctors who follow the NUCCA Protocol.^7^, ^8^, ^9^ Thus, agreement on radiographic analysis should be studied and quantified. Therefore, the purpose of this study was to investigate the inter-examiner agreement between 2 NUCCA board-certified doctors in marking and analyzing conventional orthogonal radiographic film sets. The hypothesis was that 2 chiropractic doctors certified in the NUCCA protocol should be able to reach good inter-examiner agreement to analyze radiographs.

## Methods

### Study Design

This reliability study was designed to minimize the following 3 possible sources of variability: biological variation in a source, procedural variation, and inter-observer variability.^10^

### Ethics

The study protocol was approved by Sherman College of Chiropractic Institutional Review Board (#ERAOR083111).

### Sample Size

The requisite sample for the number of films to be analyzed in this study was determined using the WINPEPI (PEPI-for-Windows) PAIRSetc I2 computer program^11^ based on ICC within the range of 0.4 to 0.7 considered to be acceptable agreement.^11^^,^^12^ A sample of 100 to 250 paired radiographs was required to derive a 95% CI for the intraclass correlation coefficients (ICC), with a 0.10-unit margin of error for ICC within the range of 0.4 to 0.7. The first set of 150 paired radiographs was to be analyzed and statistically evaluated in order to refine the sample size needed for the next phase to reach the target goal of a 95% CI for the ICC, with a 0.10-unit margin of error for ICC within the range of 0.4 to 0.7.

### Study Preparation

**Fig 1:**
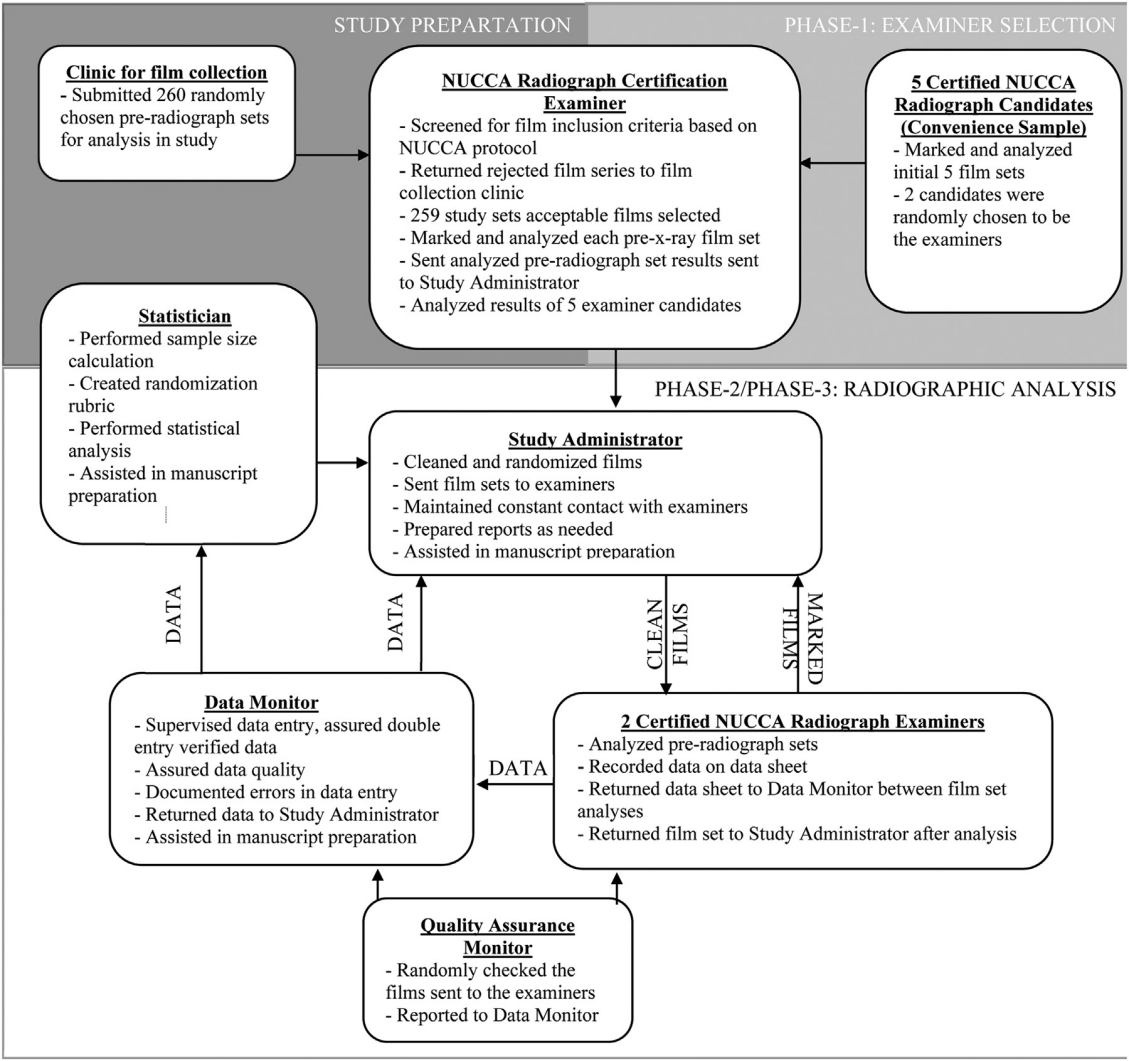
Study flowchart.

The study was developed following the NUCCA guidelines for radiographic marking.^7^ Previously taken radiographs were gathered from the office records of a single board-certified NUCCA doctor to reduce variability between film sets. The NUCCA standard for taking radiographic images includes the use of special lead filters to reduce patient radiation exposure.^7^^,^^13^ Inactive patient film files were gathered, and 260 patient film files were submitted for the study. The NUCCA certification and standards board co-chair reviewed these films, selecting only those of sufficient quality. Of the 260 film sets submitted, 259 were deemed suitable for the study (Fig 1). The films were sent to the data manager, who removed all pencil markings and randomized them.

### Phase 1: Examiner Training and Selection

Five board-certified doctors volunteered for 2 study examiner positions. A 2-hour training session was held at 2 biannual NUCCA conferences to clarify the study procedure points selected for measurement. One method was agreed upon and reviewed with examiners to standardize the process. Upon training conclusion, 5 sets of films were sent out to the candidates to determine if they agreed on the analysis. All 5 doctors agreed on the analysis; however, 1 doctor dropped out due to personal time commitments, resulting in 4 equally qualified candidates. Of the qualified candidates, 2 examiners were randomly selected.

### Phase 2: Initial Measurements

The 2 selected examiners were scheduled to complete the analysis of 150 film sets. The examiners marked the radiographs and calculated the atlas laterality and rotation. The examiner recorded the measurements on both the radiographs and on a sheet of paper that was placed in the film jacket with the radiographs. An independent data manager received the examiner analysis and supervised double-entry verification into a study-specific Excel spreadsheet (Microsoft Corporation, Redmond, Washington). The study administrator was responsible for film set randomization and removing pencil marks between observations, assuring clean, unmarked films were sent to examiners for analysis. Quality assurance checks of film packages by an outside examiner were used to screen for deficiencies in films being sent out.

### Phase 3: Remaining Measurements

After phase 2, examiners discussed implementation of the analysis protocol and discovered some inconsistencies. Some radiographs submitted for the study were missing right and left identification (ID) markers. If an examiner reversed a film on their analysis compared to the other examiner due to missing right or left markers, the resulting misalignment degree measurement would be the same, but the side determined could be the opposite. For phase 3, examiners agreed to always place the ID blocker on the left side of the view box.

**Fig 2:**
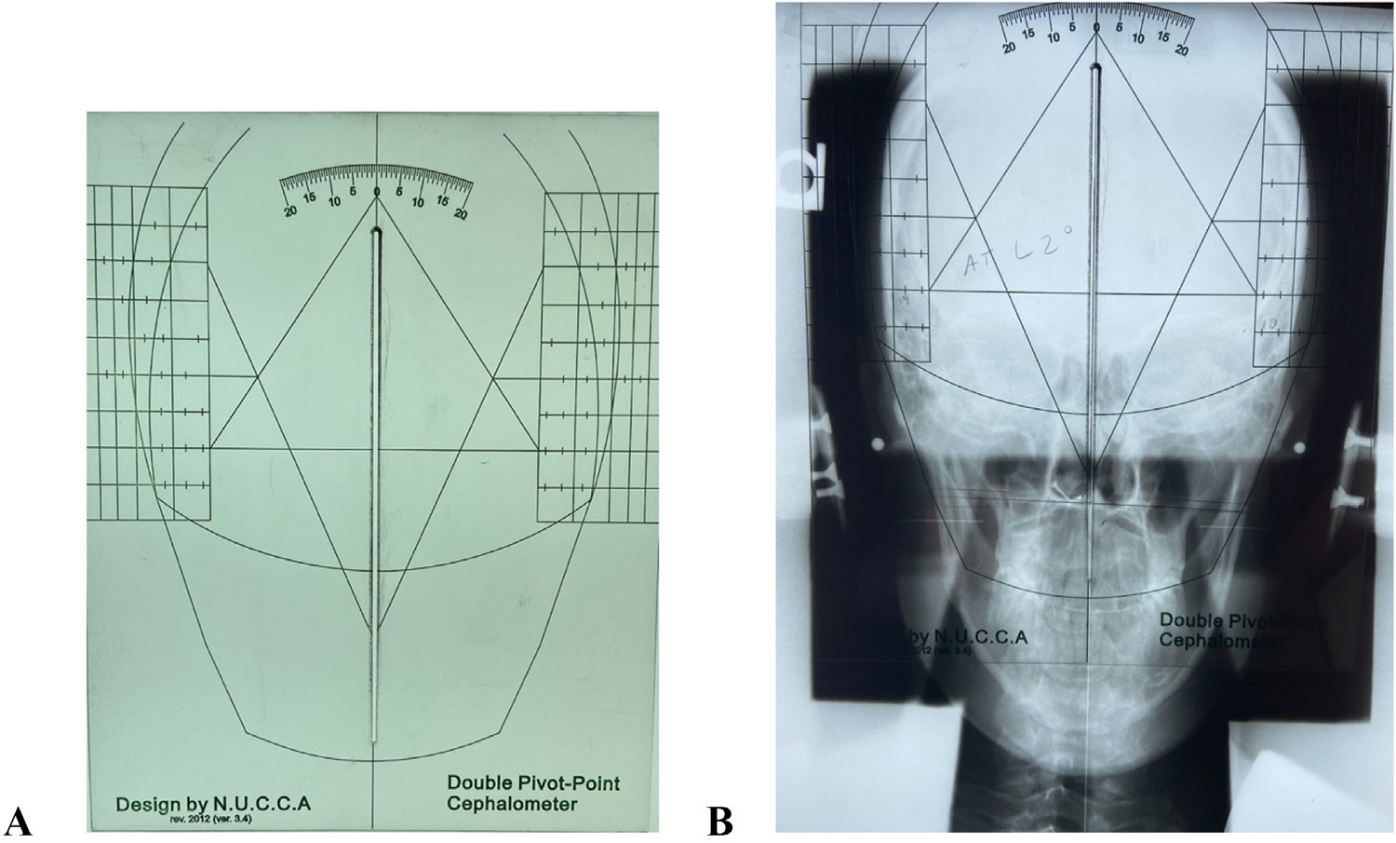
A) The NUCCA double pivot point cephalometer, which is used to divide the skull. B) An example of the cephalometer when overlaid on a plain film nasium radiograph.

There were 2 different models of the plexiglass NUCCA cephalometer tools (Fig 2), so duplicate toolsets were mailed to each of the examiners to ensure the same model was utilized. Examiners also agreed on how much of the parietal portion of the skull to analyze on the nasium film. Further, an agreement between the examiners was made about the anatomical points to be used for the exact identification and construction of the central skull line, which is crucial for excellent analysis agreement.

Phase 3 was conducted the same way as phase 2 on the remaining 104 paired radiographs. Allowing examiners to converse about the previously analyzed films could have introduced a bias that affected agreement. To mitigate bias, films utilized in phase 1 and phase 2 were not used again. The goal was to maintain optimal continuity of marking and measuring films so that the results reflected doctor analysis only and not procedural variances.

### Description of Measurements

#### Atlas Laterality

**Fig 3:**
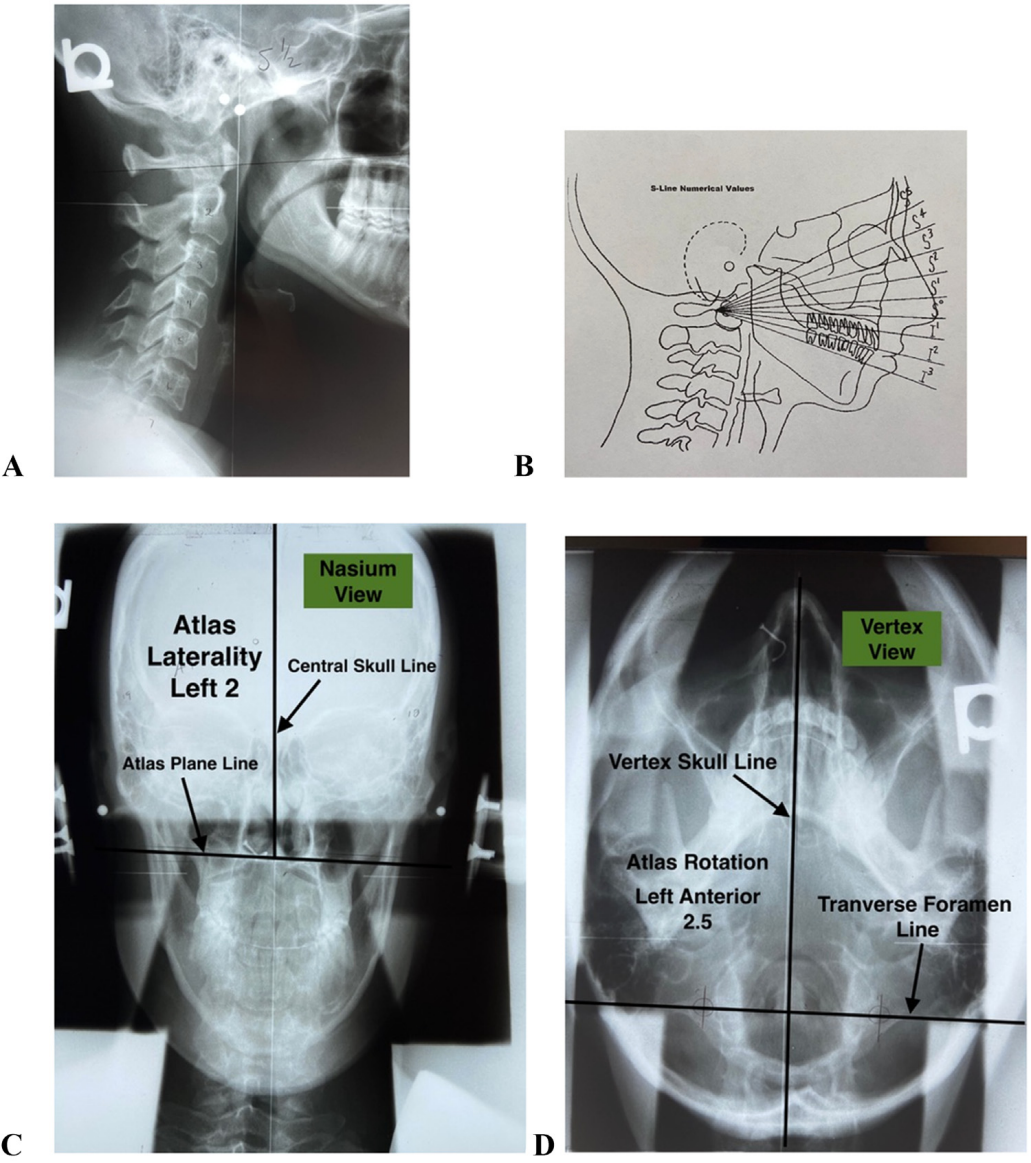
The lateral view (A) is used to measure the S-line (B) to acquire the nasium view. The nasium (C) measures atlas (C1) laterality and the vertex (D) measures atlas (C1) rotation. Fig 3C and 3D illustrate the measurements used by both examiners in the study.

The nasium view (Fig 3C) is taken based upon an “S-Line” measurement (Fig 3B) from the lateral radiograph (Fig 3A). Measurements taken from the nasium view may show lateral displacement of the first cervical vertebrae or the atlas (C1) around the condyle of the occipital bone and is determined by measuring an angle between the atlas plane line and central skull line.^7^

The atlas plane line represents the position of C1 as a measurement in the frontal plane relative to the horizon. It is constructed by locating 2 points where each of the atlas transverse processes intersect with each of the lateral masses. Once the points are determined, a line is drawn between the points and extended to the edge of the film side to side to form the atlas plane line.^7^

The central skull line is constructed by locating the median plane of the cranial vault. This is done by utilizing a NUCCA cephalometer (Fig 2). The instrument is placed over a nasium radiograph, and the grid lines imprinted on the cephalometer bisect the skull. The opening in the center of the instrument allows a sharp pencil to make marks. The process is repeated by moving the instrument up and down the skull and matching the gridlines to capture multiple center-line points. The points connect and become the central skull line.

Once the atlas plane line and the central skull line have been drawn, the acute angle between the 2 lines is measured. The acute angle is considered the side of atlas laterality. Once the atlas laterality is determined, the number of degrees is written on the acute angle side (either right or left) (Fig 3C).

#### Atlas Rotation

The vertex film gives the degree and direction of forward or backward movement of the atlas in the transverse plane (Fig 3D). This is known as atlas rotation and is recorded as anterior or posterior relative to the side of atlas laterality.^7^

The transverse foramina of C1 are divided into quadrants, and the center point of each foramen is marked. A line is drawn through the centers of the foramina and extended to the edge of the film side to side to form the transverse foramen line.^7^

The vertex skull line is marked by placing dots along lateral parts of the nasal opening, and then a center point between the 2 points is marked. Dots are then placed at the lateral border of the second cervical vertebrae (C2) odontoid where it attaches to the C2 vertebral body. A point is found and marked for the C2 odontoid center. A line is drawn between the nasal center point and the C2 odontoid center, which is the vertex skull line.^7^

A protractor measures the angle between the vertex skull line and the transverse foramen line. The side of atlas laterality is taken from the nasium film, and the rotation is measured either anterior or posterior relative to the side of atlas laterality. When the angle is measured as an acute angle on the side of laterality, it is labeled anterior rotation. When the angle measured is an obtuse angle on the side of atlas laterality, it is labeled posterior rotation. The total number of degrees anterior or posterior is listed on the vertex radiograph (Fig 3D).^7^

### Statistical Analysis

Data management and analysis were performed using Predictive Analytics Software (SPSS, version 22; IBM Corporation, Armonk, New York). Using data from phase 2 (measurements from 150 film sets), it was projected that examiners could analyze film sets in determining atlas laterality and atlas rotation with at least 90% agreement on side atlas laterality and atlas rotation within one-half a degree, with the ICC >0.7. Measurements were used at 0.25°, reflecting the NUCCA standards, which utilize 0.25° increments in calculations and measurements. Sample size calculations were based on achieving ICC values of 0.4 to 0.7. Assuming an ICC of 0.7, 101 paired observations were needed to derive a 95% CI with a 0.1-unit margin of error. ICCs were rated as the following: poor to fair (below 0.4), moderate (0.41-0.60), excellent (0.61-0.80), and almost perfect (0.81-1).^12^ The required sample size ranged from 150 to 254 paired observations. Following phase 2, an additional 104 film sets were analyzed, resulting in a total of 254 measurements.

The direction of atlas laterality, originally captured as left or right, was coded for statistical analysis purposes as negative or positive, respectively. The direction for the atlas rotation, originally captured as anterior or posterior (relative to the side of atlas laterality), was coded for statistical analysis purposes as negative or positive in the transverse plane. If the examiners disagreed on side of laterality but agreed on the side of rotation, then 1 doctor could have called it a left laterality with an anterior rotation, and the other doctor would have called it a right laterality with a posterior rotation. Coding the atlas rotation as positive or negative in the transverse plane gave it an “absolute value” that did not correspond to the side of atlas laterality. Thus, each recorded measurement was entered as a positive or negative number, with the sign representing the direction and the number representing the degree of atlas laterality or rotation. The ICC from the 2-way mixed model for absolute agreement with corresponding 95% CI was used as the primary outcome measure to assess the level of absolute agreement (inter-rater agreement) between the measurements of the 2 examiners.^14^^,^^15^ As typical in the analysis of agreement between 2 quantitative measurements, the Bland-Altman plot analysis^16^ was used as an alternate method for assessing agreement in the 2 sets of measurements, allowing for the assessment of any systematic difference and potential proportional bias. Statistical significance was assessed at the 5% level of significance. The method in the present study compared the mean absolute measurement for laterality and rotation to the range of error at which approximately 80% of the cases showed agreement between examiners.

## Results

There was 96.1% (244/254) agreement between examiners on the side of atlas laterality and 94.5% (240/254) agreement on direction of atlas rotation (n = 254). In phase 2, the agreement was 94.6% (142/150) for laterality and 92.0% (140/150) for rotation (n = 150). In phase 3, laterality agreement was 98.1% (102/104), and rotation agreement was 96.1% (100/104).

**Fig 4:**
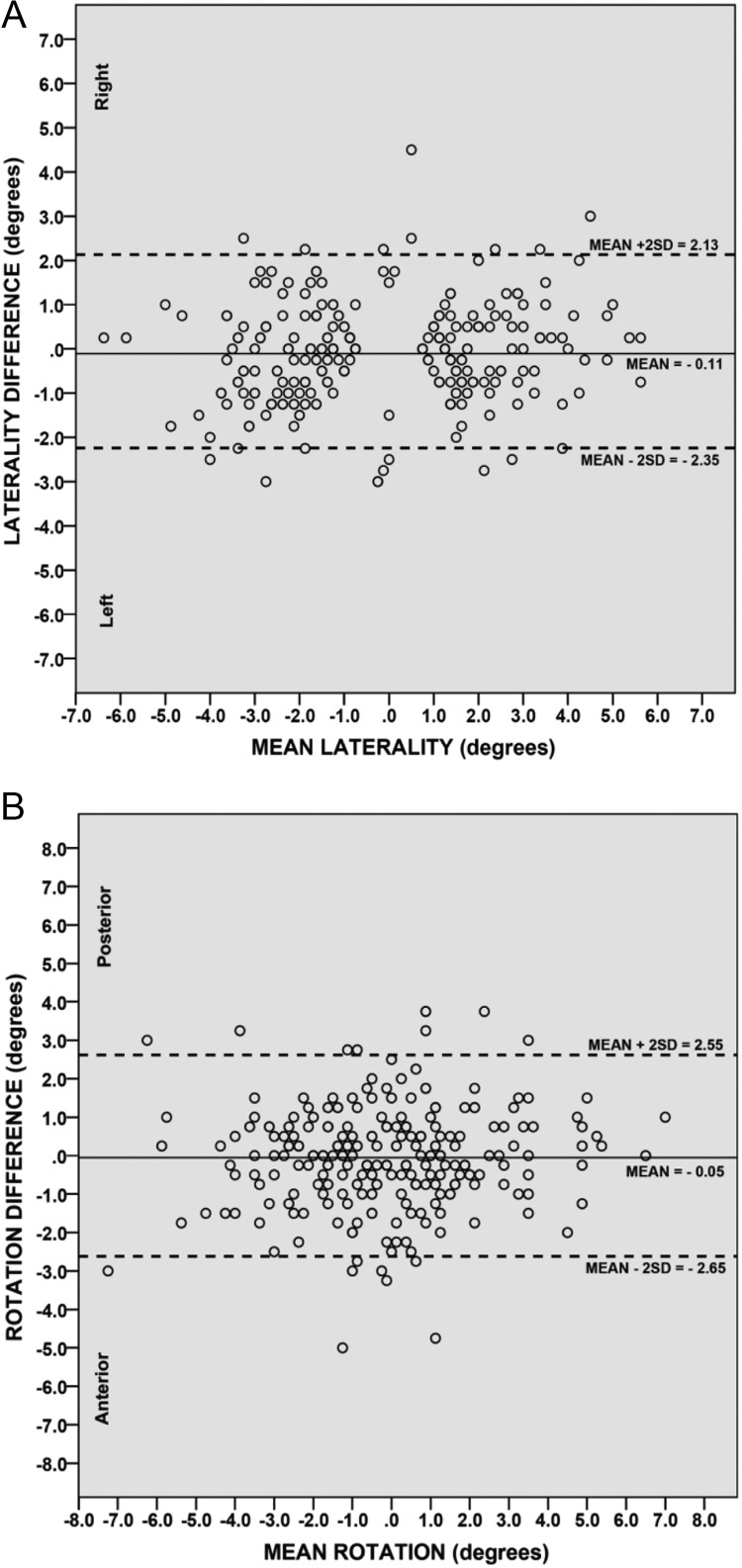
Bland-Altman agreement plots (n = 254). Atlas laterality (A). Atlas rotation (B). An indicator of excellent agreement, these Bland-Altman plots show only 15 out of 254 points outside the agreement limits of mean +2 standard deviations. The horizontal (x) axis represents the average of the 2 examiners’ measurements of atlas laterality and rotation. The vertical (y) axis represents the difference between the measurements of the 2 examiners shown outside the 95% agreement limits. The laterality plot shows 15 out of 254 points, or 5.9% of measurements. The atlas rotation plot shows 16 out of 254 points, or 6.2% of measurements outside the agreement limits.

Using all 254 sets of measurement, the ICC was 0.95 (95% CI, 0.93-0.96) for laterality and 0.92 (95% CI, 0.89-0.94) for rotation. The mean difference in the measurements of atlas laterality between the 2 doctors was −0.11, *P* = .12 and 0.05, *P* = .55 for atlas rotation, both not significantly different from zero and thus indicative of good agreement. Visual examination of the Bland-Altman plots was not suggestive of any proportional bias in the 2 measurements of atlas laterality (Fig 4A) and atlas rotation (Fig 4B), as there was no immanent trend of points above or below the mean difference line. Only 5.9% (15/254) and 6.2% (16/254) of the points were displayed outside the 95% confidence limits of agreement for atlas laterality and rotation, respectively.

A range of error of 1.25° for atlas laterality was observed at approximately 80% agreement of cases (in 207 of the 254 total cases = 81.5%), which was less than the 2.18° for mean absolute laterality. For rotation, a range of error of 1.50° was observed at 82.7% of the cases, which was also less than 1.85° for mean absolute rotation. Table 1 shows the results of the measurements of atlas laterality.

**Table 1:** Examiner Agreement Intervals

Examiner Agreement Interval	No Examiner Error	Percentage
0.25	75	29.5
0.5	112	44.1
0.75	152	59.8
1	180	70.9
1.25	207	81.5
1.5	220	86.6
1.75	231	90.9
2	235	92.5
2.25	242	95.3
2.5	247	97.2
2.75	249	98.0
3	253	99.6
4	254	100.0

Examiner agreement of atlas laterality by intervals of 0.25°.

## Discussion

This is the first study utilizing a statistically significant sample size to evaluate the agreement of 2 board-certified NUCCA doctors in radiographic analysis of the upper cervical spine. Examiner ICCs in this analysis of orthogonal radiographs displayed almost perfect agreement.^12^ This study showed that the range of error was smaller than the mean absolute degree of laterality and rotation, suggesting that the measurements identified real degrees of atlas misalignment with a high level of agreement.

The discussions between phases 2 and 3 provided measurable improvements in measurement agreement between examiners. The changes made from phase 2 to phase 3 led to a 4% increase in agreement, as the ICCs improved to 0.94 and 0.96 for the atlas rotation and laterality, respectively. This reliability study demonstrated the value of ongoing training and continuous feedback to the chiropractic practitioner on radiographic analysis results. Future practitioners would benefit from regular evaluation and feedback on their radiographic examinations.

Of other studies looking at reliability of upper cervical marking systems, 1 did not agree with the current.^1^ Sigler and Howe used 20 film sets in their study, and they collected them from 2 different offices (10 each). They had 3 examiners mark each of the 20 films and compare them. The weakness in their study, as pointed out by Jackson, was the inappropriate use of Bartko's formula in relation to the ICC.^3^ The present study used Bland-Altman in conjunction with ICC for data analysis and used a large sample size of 254 films.

### Limitations

The relevance of atlas misalignment is a topic of importance for further study; however, the current study aimed to investigate examiner agreement only. Film selection for this study may not reflect clinical practice since doctors in practice do not get to actively select films based on quality or anatomical anomalies.^1^^,^^4^ This study used a convenience sample of radiographs with optimized quality intended to reduce variability, and all radiographs used were of acceptable quality for NUCCA board-certification films. Thus, this controlled environment does not necessarily reflect actual practice. The 2 examiners used in the study were trained and selected and thus do not necessarily represent all NUCCA practitioners.

### Future Studies

Future studies could consider using a random sample of films or consecutive films, a larger and random sample of NUCCA-certified doctor-examiners, and films showing no misalignment as a control group, and they could allow examiners to review the acceptability of film sets used in the study, as suggested by Hubbard.^4^

## Conclusion

This investigation of the inter-examiner agreement between 2 NUCCA practitioners in marking and analyzing conventional orthogonal radiographic film sets showed almost perfect agreement and no immanent proportional bias. The range of error was smaller than the mean absolute degree of measured laterality and rotation.

## Acknowledgments

The authors thank Jack Stockwell, Kathy Waters, Vivek Soham, and Nina Pavlovic for their expertise and support of the completion of this project.

## Funding Sources and Conflicts of Interest

The following entities funded this study: Tao Foundation, Calgary, Alberta, Canada; Upper Cervical Research Foundation, Monroe, Michigan; and the Ralph R. Gregory Memorial Foundation, Alberta, Canada. No conflicts of interest were reported for this study.

## Contributorship Information

Concept development (provided idea for the research): M.D., C.L.

Design (planned the methods to generate the results): M.D., C.L.

Supervision (provided oversight, responsible for organization and implementation, writing of the manuscript): M.D., J.L.D, D.G.H.

Data collection/processing (responsible for experiments, patient management, organization, or reporting data): M.D., J.F.H.

Analysis/interpretation (responsible for statistical analysis, evaluation, and presentation of the results): J.L.D., D.G.H., J.F.H., H.N.

Literature search (performed the literature search): J.L.D., J.F.H.

Writing (responsible for writing a substantive part of the manuscript): J.L.D., J.F.H., D.G.H., H.N.

Critical review (revised manuscript for intellectual content, this does not relate to spelling and grammar checking): J.L.D., D.G.H., J.F.H., H.N.

**Practical Applications**

• We measured the inter-examiner agreement between radiograph markings of 2 National Upper Cervical Chiropractic Association board-certified chiropractors.
• There was 96.1% agreement between the examiners.
• We found no apparent proportional bias.

Alt-text: Unlabelled box
